# Supplementary material for: Chromothripsis during telomere crisis is independent of NHEJ, and consistent with a replicative origin
Source: Genome Res. 2019 May;29(5):737–49. doi: 10.1101/gr.240705.118 (PMC6499312; doi:10.1101/gr.240705.118)
Supplement: Supplemental Material [file supp_gr.240705.118_Supplemental_file_1.zip › contigs/annotated_contigs/DB57/contig.2.DB57_length_627_mean_cov_7.60765550239.docx]

**DB57_length_627_mean_cov_7.60765550239**

```
AATGGTGGCCACAGGGCATGGATCCCCAGAGGCACTCATCTCCGTCAGAGGCCAGATCCTATCTCTTTCTTTCTAGATGTTTGAGCAGG
 >chr7:2464871-2465185 - E=3e-177
GCCATCTCATAGGCCCTTTTGGGGGTGGAGGGTAAAAGGTGAATATGAAATATATCCATTTTCAGTGGCTATGACCTGGTCTGGGGTTT

CTGGAAGAGCCCAACAGTCTGGACCCCTCTCTCCTGGCACTCACCCTTGTCTGCACTGTGGTTGTGTCCACACCTGTCTTGACCGACAT

GCTGACTAAGCGCGGTCCTCACTGGGCCGGGGTTTGTTTTTTTAC|GT|AGATAACCTGAGGTCAGGAGTTAGAGACCAGCCTGGCCAA
                                              >chr7:2600593-2600836 - E=2e-134
CATGGTGAAACCCCACCTCTACTAAAAATAGAAAAATTAGCTGGGTGTGGTGGCAGGCGCCTGTAGTCCCAGCTACTGAGGAGGCTGAG

GCAGGAGAATCCTTTGAACCTGGGAGGCAGAGACTGCAGTGATCTGAGATGGCATCATTGCACTCCAGCCTGGGTGGCAAAGCGAGACT

GTGTCTTTTTTTTTTTTTTTTTT|AGACATATTCTCTCTCTTCTGCTATGCGTGAGTTCCTTTGTACTTTCTTTGCTCGCTGCCATCTC

CTCCTCC
```
